# Homologous Recombination-Independent Large Gene Cassette Knock-in in CHO Cells Using TALEN and MMEJ-Directed Donor Plasmids

**DOI:** 10.3390/ijms161023849

**Published:** 2015-10-09

**Authors:** Tetsushi Sakuma, Mitsumasa Takenaga, Yoshinori Kawabe, Takahiro Nakamura, Masamichi Kamihira, Takashi Yamamoto

**Affiliations:** 1 Department of Mathematical and Life Sciences, Graduate School of Science, Hiroshima University, Hiroshima 739-8526, Japan; E-Mail: x000906z@hiroshima-u.ac.jp; 2 Department of Chemical Engineering, Faculty of Engineering, Kyushu University, Fukuoka 819-0395, Japan; E-Mails: kawabe@chem-eng.kyushu-u.ac.jp (Y.K.); kamihira@chem-eng.kyushu-u.ac.jp (M.K.); 3 Faculty of Agriculture, Kyushu University, Fukuoka 812-8581, Japan; E-Mail: tnaka@agr.kyushu-u.ac.jp

**Keywords:** TALEN, gene knock-in, CHO cells, microhomology-mediated end-joining

## Abstract

Gene knock-in techniques have rapidly evolved in recent years, along with the development and maturation of genome editing technology using programmable nucleases. We recently reported a novel strategy for microhomology-mediated end-joining-dependent integration of donor DNA by using TALEN or CRISPR/Cas9 and optimized targeting vectors, named PITCh (Precise Integration into Target Chromosome) vectors. Here we describe TALEN and PITCh vector-mediated integration of long gene cassettes, including a single-chain Fv-Fc (*scFv-Fc*) gene, in Chinese hamster ovary (CHO) cells, with comparison of targeting and cloning efficiency among several donor design and culture conditions. We achieved 9.6-kb whole plasmid integration and 7.6-kb backbone-free integration into a defined genomic locus in CHO cells. Furthermore, we confirmed the reasonable productivity of recombinant scFv-Fc protein of the knock-in cells. Using our protocol, the knock-in cell clones could be obtained by a single transfection and a single limiting dilution using a 96-well plate, without constructing targeting vectors containing long homology arms. Thus, the study described herein provides a highly practical strategy for gene knock-in of large DNA in CHO cells, which accelerates high-throughput generation of cell lines stably producing any desired biopharmaceuticals, including huge antibody proteins.

## 1. Introduction

Designable and controllable knock-in of large gene cassettes has been long desired for animal cell engineering, including production of biopharmaceutical proteins by Chinese hamster ovary (CHO) cells [1]. Recent advancement of genome editing technologies has facilitated an efficient gene knock-in in various cultured cells [2], mainly based on the enhancement of homologous recombination (HR). However, targeting vectors for HR-mediated gene knock-in require long homology arms corresponding to each genomic target site, which makes it difficult to lower the labor and enhance the throughput. On the other hand, non-homologous end-joining (NHEJ)-dependent gene knock-in methods have been reported by several groups [3,4,5,6,7]. NHEJ-based methods not only reduce the labor of constructing targeting vectors, but also overcome the difficulty of gene knock-in in cells and animals with low HR frequency. Despite these advantages, however, NHEJ-based methods also have several disadvantages such as co-delivery of plasmid backbone sequence and uncontrollable knock-in directions and junctions.

We have recently reported an alternative strategy for gene knock-in, termed the PITCh (Precise Integration into Target Chromosome) system [8], which has the best of both worlds, *i.e.*, (1) the system does not rely on HR; (2) long homology arms are thus not needed; (3) long-range PCR is also not needed for genotyping; (4) backbone-free integration can be performed; and (5) knock-in directions and junctions are controllable. The PITCh system utilizes microhomology-mediated end-joining (MMEJ) instead of NHEJ or HR, resulting in precise gene knock-in through extremely short microhomologies (≤40 bp). Since these microhomologies can easily be added by PCR or insertion of synthesized oligonucleotides, the construction of the donor vector for the PITCh system (PITCh vector) does not require PCR amplification of genomic DNA sequence. Using the PITCh system, we have succeeded in efficient gene knock-in in human cells such as HEK293T and HeLa cells and animal organisms such as frogs, zebrafish, and silkworms [8,9].

In CHO cells, programmable nuclease-mediated gene knock-in has mainly been performed by NHEJ-dependent methods, because they have regarded as one of the HR-inactive cells [3,4,7]. Recently, Kildegaard and colleagues reported successful gene knock-in via HR in CHO cells [10]. However, all the previous reports were proof-of-concept studies by knocking-in reporter genes such as fluorescent protein genes and drug selection markers. In addition, the capability of programmable nuclease-mediated knock-in of the large DNA around 10 kb was never validated. Here, we report the purposeful examples of knocking-in the large DNA using transcription activator-like effector nuclease (TALEN) and the PITCh system, carrying a single-chain Fv-Fc (*scFv-Fc*) cassette, along with *DsRed* and puromycin resistance gene (*PuroR*) cassettes. Our protocol enables rapid construction of donor vectors, simple genotyping with fluorescence observation and easy PCR screening with short-range amplification, and efficient isolation of knock-in cell clones by a single limiting dilution using a 96-well plate.

## 2. Results and Discussion

### 2.1. Design and Activity Validation of TALEN Targeting the HPRT1 Gene

**Figure 1:**
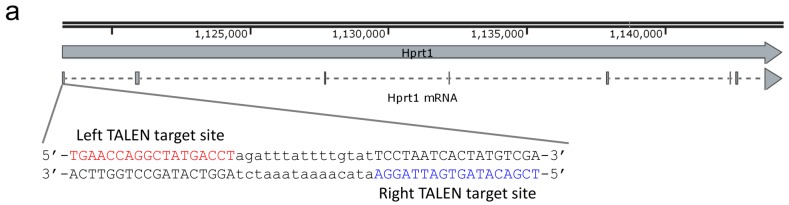

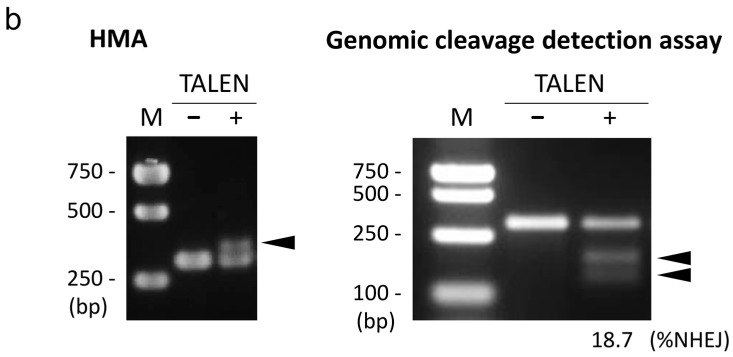
Design of TALEN and validation of its activity. (**a**) Schematic illustration of the TALEN target site. The genomic context was visualized using a SnapGene Viewer software (Chicago, IL, USA) (http://www.snapgene.com/), incorporating a GenBank-formatted NCBI data [19]. Solid line arrow indicates *HPRT1* gene. Dash line arrow indicates *HPRT1* mRNA. Red and blue letters indicate the left and right TALEN target sites, respectively. Lowercase letters indicate spacer sequence; (**b**) Electrophoretic gel images of heteroduplex mobility assay (HMA) and genomic cleavage detection assay. An arrowhead in the left panel shows the up-shifted heteroduplex band. Arrowheads in the right panel show the digested fragments. M, Wide-Range DNA Ladder (100–2000 bp) (TAKARA BIO INC., Shiga, Japan). The percentage of non-homologous end-joining-dependent mutations (%NHEJ) was estimated using an ImageJ software (Bethesda, MD, USA) (http://imagej.nih.gov/ij/) according to the previous report [18].

According to the very recent report, clustered regularly interspaced short palindromic repeats (CRISPR)/CRISPR-associated protein 9 (Cas9)-mediated gene knock-in resulted in off-target integration of donor DNA as well as on-target integration in CHO-K1 cells [7]. Furthermore, CRISPR/Cas9 reportedly induces relatively high off-target mutations in some cultured cells [11,12,13]. We therefore chose TALEN to induce site-specific DNA double strand break (DSB) at the targeted locus. To obtain an optimal level of exogenous gene expression, we have chosen the hypoxanthine phosphoribosyltransferase 1 (*HPRT1*) locus, which was previously demonstrated to be an ideal locus for stable expression of exogenous gene cassettes in human fibrosarcoma cells [14]. We designed and constructed TALEN against *HPRT1* gene in CHO-K1 cells. The TALEN target site was determined using a partially annotated genomic sequence (NCBI Gene ID: 100769768). The most 5′ exon was used for the targeting, although it was supposedly not an actual first exon (Figure 1a). Subsequently, we evaluated the genome editing efficiency by heteroduplex mobility assay (HMA) and genomic cleavage detection assay. The HMA is the simplest method for detecting programmable nuclease-induced mutations, by electrophoresing the PCR products amplified around the target site [15,16]. The genomic cleavage detection assay, also known as Surveyor assay or Cel-I assay, is a method using a mismatch-sensitive endonuclease [17]. The mutagenic efficiency can be calculated by densitometric analysis of an undigested band and digested bands [18]. We transfected TALEN expression vectors into CHO-K1 cells and evaluated the targeted DSB induction by these two methods (Figure 1b). The HMA showed that the heteroduplex band was clearly observed only in TALEN-introduced sample. The mutation frequency was quantitated as 18.7% by the genomic cleavage detection assay. These results suggest that the constructed TALEN can cleave the genomic *HPRT1* locus in CHO-K1 cells.

### 2.2. Gene Knock-in into the HPRT1 Locus Using the PITCh System

#### 2.2.1. Whole Plasmid Integration Carrying *DsRed* and *PuroR* Gene Cassettes

**Figure 2:**
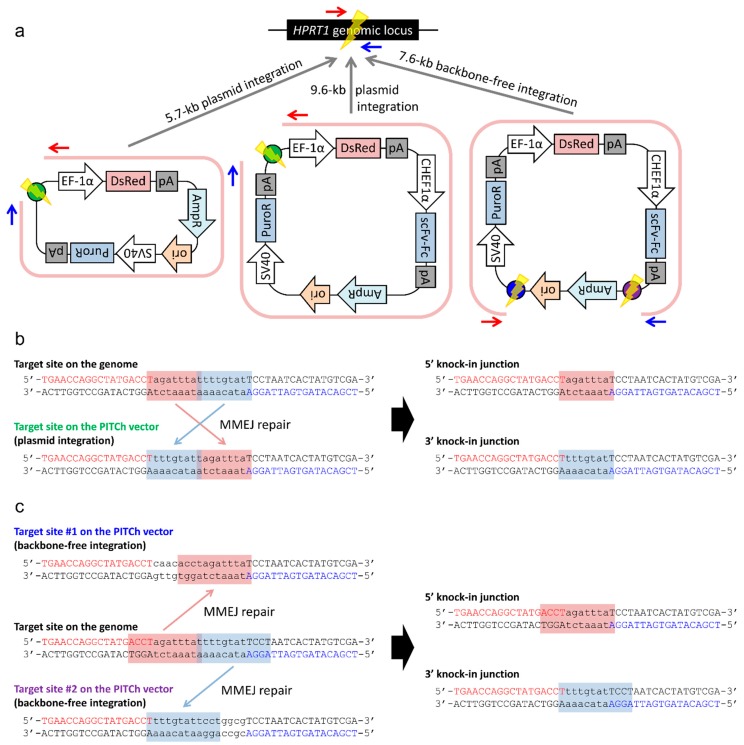
Schematic illustration of PITCh vectors and TALEN target sites. (**a**) Summary of the three kinds of PITCh vectors and knock-in experiments. Green, blue, and purple circles indicate TALEN target sites on the PITCh vector, related to (**b**,**c**). Yellow shapes indicate DNA double strand breaks. Pink lines indicate the regions that are expected to be knocked-in. Red and blue arrows indicate the positions of primers for the amplification of 5′ and 3′ junctions, respectively, related to Figure 3; (**b**,**c**) The TALEN target sites and knock-in junctions of plasmid integration (**b**) and backbone-free integration (**c**). Positions of each target site on the PITCh vector was shown in (**a**). Pink and blue boxes indicate designed microhomologies. Red and blue letters indicate the left and right TALEN target sequences, respectively. Lowercase letters indicate spacer sequence.

To estimate the capacity and efficiency of gene knock-in at the *HPRT1* locus in CHO-K1 cells, we first performed whole plasmid integration using the TALEN-mediated PITCh system, which was previously proven to work well in human cells [8]. *DsRed* and *PuroR* gene cassettes, driven by elongation factor 1 α (EF-1α) and Simian virus 40 (SV40) promoters, respectively, were independently placed in the donor plasmid, to easily screen the donor-incorporated cells (Figure 2a). Importantly, however, these gene cassettes can work even if the plasmid is integrated in the genome via random integration. Subsequently, a modified TALEN target sequence, enabling MMEJ-mediated PITCh, was added in the donor plasmid. The modified TALEN site contains different spacer sequence from the original target site on the genome, as shown in Figure 2b. After the occurrence of DSBs at each target site, ideally 9-bp microhomologies can be utilized for MMEJ-mediated integration (Figure 2b).

**Figure 3:**
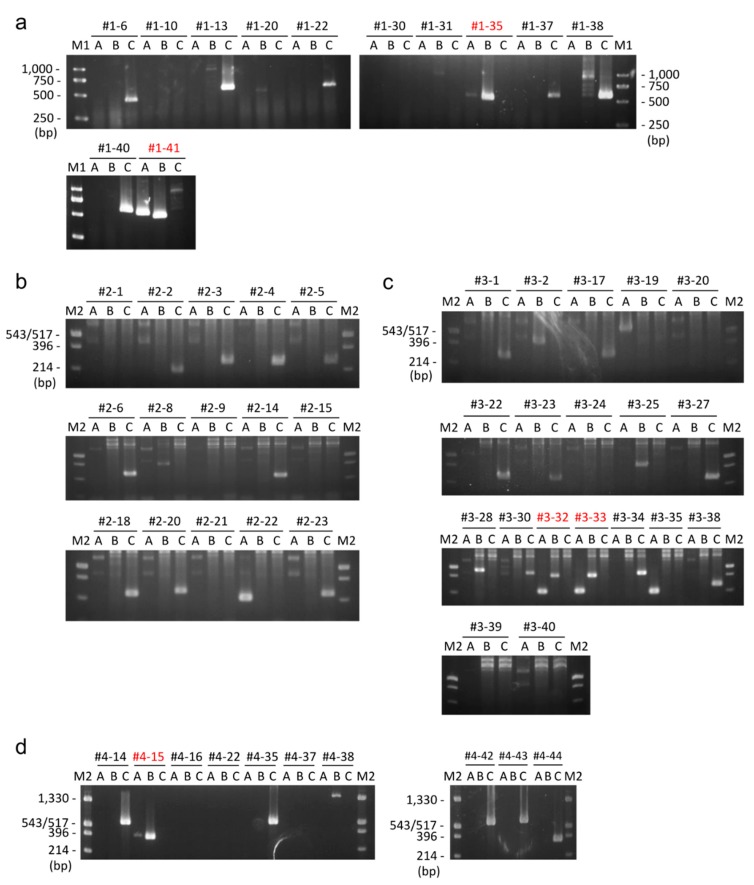
PCR genotyping. The results were shown separately for whole plasmid knock-in without carrying an *scFv-Fc* cassette (**a**), whole plasmid knock-in carrying an *scFv-Fc* cassette with puromycin selection from 72 h post-transfection (**b**) or from 24 h post-transfection (**c**), and backbone-free knock-in (**d**). The clone IDs are shown at the top of each panel. Red letters indicate correctly knocked-in clones. A, B, and C indicate the PCR products of 5′ junction, 3′ junction, and non-knock-in allele, respectively. M1, Wide-Range DNA Ladder (100–2000 bp) (TAKARA BIO INC.). M2, pUC/HinfI.

**Figure 4:**
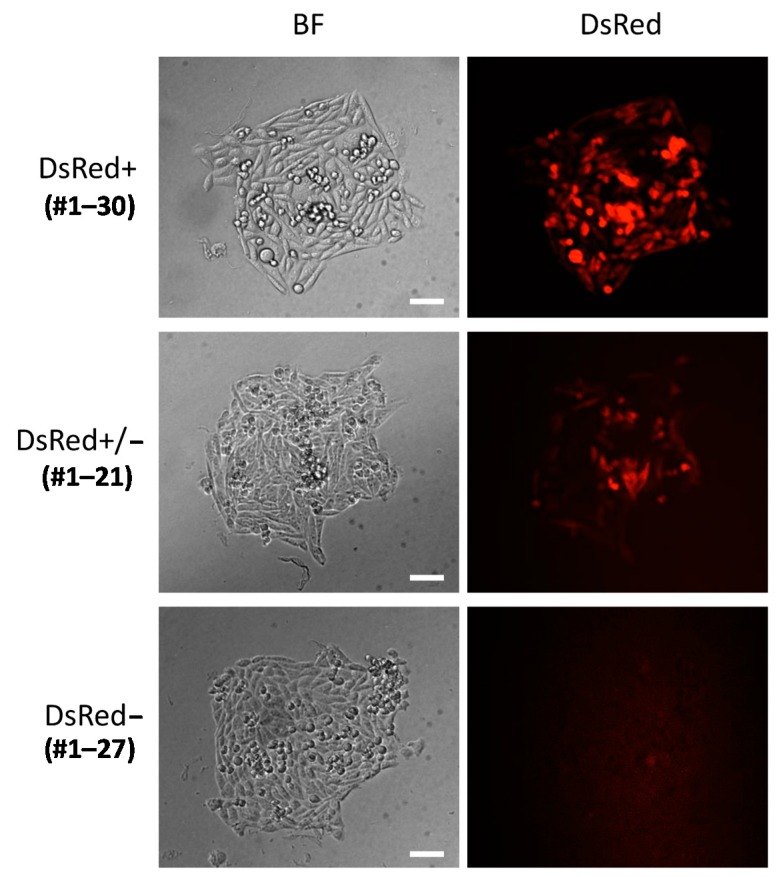
Examples of bright-field and fluorescence images. BF, bright field. Bars, 30 μm.

**Figure 5:**
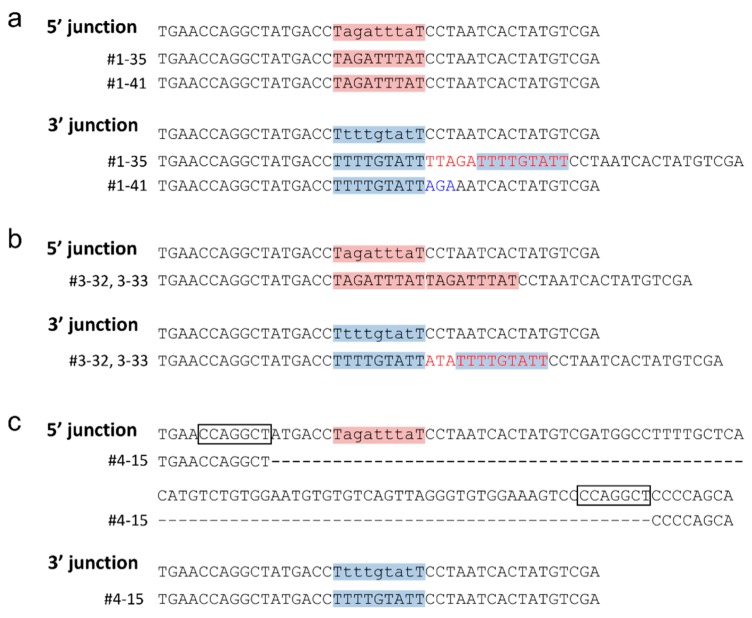
Sequences of 5′ and 3′ junctions of knock-in cell clones. The results were shown separately for whole plasmid knock-in without carrying an *scFv-Fc* cassette (**a**), whole plasmid knock-in carrying an *scFv-Fc* cassette with puromycin selection from 24 h post-transfection (**b**), and backbone-free knock-in (**c**). Intended knock-in junctions are shown at the top of each panel. Pink and blue boxes indicate designed microhomologies. Black boxes indicate accidentally existed microhomologies. Red letters indicate insertions. Blue letters indicate substitutions.

The donor vector was transfected into CHO-K1 cells along with the left and right TALEN plasmids. After approximately one week of puromycin selection from 72 h post-transfection, single cell isolation was performed using a 96-well plate. The single cell clones were cultured for another one week, and then DsRed expression was observed using a fluorescence microscope. Of 33 colonies observed, 17, 11, and 5 colonies were evaluated as DsRed+, DsRed+/−, and DsRed−, respectively (Table 1 and Figure 4). This highly heterogenous expression pattern suggested that highly frequent random integrations have occurred, because the random integrants often display high heterogeneity in exogenous gene expression [20]. A portion of DsRed+ or DsRed+/− clones were further analyzed by PCR screening. For the PCR screening, 5′ and 3′ knock-in junctions as well as non-knock-in allele were amplified. Since *HPRT1* gene is located on the chromosome X, there only is a single copy of *HPRT1* gene in CHO-K1 cells. If the knock-in is successfully achieved, 5′ and 3′ junctions can be amplified, while no amplification can be observed for non-knock-in alleles. Of the twelve clones analyzed, two clones (#1-35, #1-41) were identified as knocked-in clones, according to the above criteria (Table 1 and Figure 3a). Sequencing analysis of 5′ and 3′ junctions revealed that the 5′ junctions were jointed via MMEJ as expected, whereas the 3′ junctions had duplicated microhomologies, presumably because of NHEJ-mediated integration, or 3-bp substitutions (Figure 5a). Although the junctions were not perfectly formed as designed, these subtle modifications are thought to have little influence on gene cassettes. Therefore, we confirmed the successful integration of the PITCh vector into the *HPRT1* locus in CHO-K1 cells.

**Table 1:** Summary of gene knock-in at the *HPRT1* locus in CHO-K1 cells.

Knock-in Type	No. of TALEN Sites on PITCh Vector	*scFv-Fc* Cassette	Knock-in Length	Puromycin Selection	No. of DsRed Expressed Clones (+; +/−; −) *	No. of Knock-in Clones/Analyzed Clones **
Whole plasmid	1	−	5.7 kb	From 72 h post-transfection	17; 11; 5	2/12 (17%)
Whole plasmid	1	+	9.6 kb	From 72 h post-transfection	7; 8; 4	0/15 (0%)
Whole plasmid	1	+	9.6 kb	From 24 h post-transfection	6; 13; 4	2/19 (11%)
Cassette (backbone-free)	2	+	7.6 kb	From 24 h post-transfection	10; 4; 15	1/10 (10%)

* All 96 wells from a single 96-well plate were screened for each experiment; ** Eleven DsRed+ cells and one DsRed+/− cells were analyzed in the first experiment. All the DsRed+ and DsRed+/− cells were analyzed in the second and the third experiments. All the DsRed+ cells were analyzed in the fourth experiment.

#### 2.2.2. Whole Plasmid Integration Carrying an *scFv-Fc* Cassette along with *DsRed* and *PuroR* Cassettes

Next, we conducted a purposeful gene knock-in carrying an independent gene cassette expressing scFv-Fc fusion protein, driven by a Chinese hamster EF-1α (CHEF1α) promoter, along with *DsRed* and *PuroR* cassettes used in the proof-of-principle knock-in (Figure 2a). The *scFv-Fc* cassette was simply added in the PITCh vector described above. The plasmid size was increased to 9.6 kb. We first performed transfection, puromycin selection, single-cell cloning, fluorescence observation, and PCR genotyping as conducted in the previous knock-in experiment. Unfortunately, however, we could not obtain any correctly knocked-in clones (Table 1 and Figure 3b), supposedly because of the larger plasmid size than the previous knock-in.

To enable stricter selection, we moved up the timing of adding puromycin from 72 h post-transfection to 24 h post-transfection. Due to this modification, we successfully identified two knock-in clones (#3-32, #3-33) by PCR screening, among nineteen clones analyzed (Table 1 and Figure 3c), although the plasmid was integrated via the NHEJ pathway (Figure 5b).

#### 2.2.3. Backbone-Free Integration Carrying *scFv-Fc*, *DsRed*, and *PuroR* Cassettes

Although we have succeeded in *scFv-Fc*-containing gene cassette knock-in, whole plasmid integration results in co-introduction of unnecessary plasmid backbone sequence. To avoid this issue, we designed another PITCh vector enabling cassette knock-in without carrying a backbone sequence. The cassette knock-in using TALEN-mediated PITCh system was discussed in the previous study [8], but no successful example has been reported yet. We added two different types of TALEN sites on the PITCh vector across the plasmid backbone (Figure 2a). The lengths of microhomologies were set to 12 bp (Figure 2c). When two DSBs are induced in this cassette-type PITCh vector, only three necessary gene cassettes can be incorporated into the *HPRT1* locus. The knock-in experiment was conducted using an improved selection strategy, settled by the plasmid integration experiments. The ten knock-in candidate clones were screened by PCR, with the result that one clone (#4-15) showed the expected band patterns (Figure 3d). We then determined the junction sequences of this clone. Interestingly, a 94-bp deletion was detected at the 5′ junction by DNA sequencing analysis, whereas 3′ junction was correctly jointed (Figure 5c). We carefully checked the 5′ junction sequence and found the fortuitously existing 7-bp microhomologies. These microhomologies were considered to be utilized for gene knock-in. In gene knockout experiments, it is well known that MMEJ-dependent deletions were frequently observed in cultured cells and animals [21,22,23]. Our finding demonstrates another interesting phenomenon particularly in HR-independent gene knock-in.

### 2.3. Confirmation of Genotype and Functionality of Knocked-in Cell Clone

**Figure 6:**
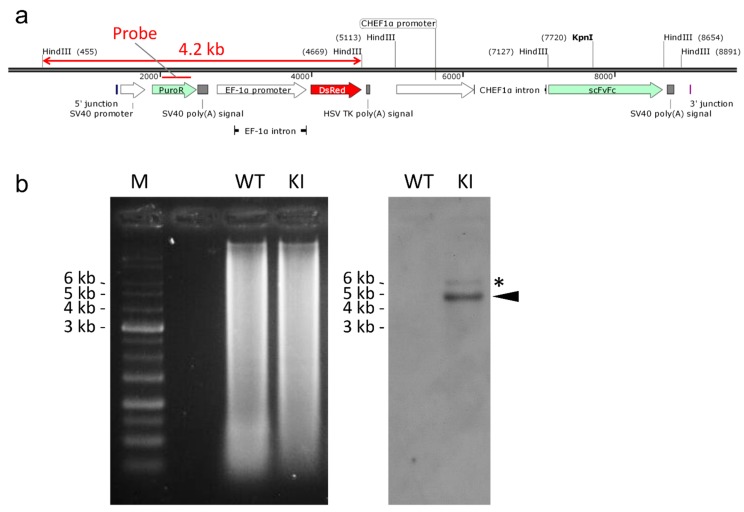
Southern blot analysis. (**a**) Schematic illustration of knocked-in allele. The genomic sequence after backbone-free integration is shown. The map was visualized using a SnapGene Viewer software; (**b**) The gel image (**left** panel) and chemiluminescent image (**right** panel) of Southern blot analysis. An arrowhead indicates the expected position of the knock-in fragment. An asterisk shows a minor band. M, 1 kb DNA Ladder (Bio Regenerations, Yokohama, Japan). WT, wild type. KI, knock-in (#4-15).

To confirm the genotype of the knocked-in cell clone, we performed Southern blot analysis using the clone #4-15, together with the wild-type CHO-K1 cells as negative control cells. The probe was designed within the *PuroR* gene in the knock-in cassette (Figure 6a). The digested genomic DNAs were electrophoresed and Southern blotting was performed accordingly. As expected, we observed no band in the wild-type lane, whereas the expected 4.2-kb fragment was clearly detected in the knock-in lane, although a faint minor band was also observed (Figure 6b). Since CHO-K1 cells have only one *HPRT1* gene, a band derived from random integration or tandem integration should be comparable in signal intensity to the 4.2-kb band, but the minor band appeared herein has much weaker signal than the expected band. Therefore, we conclude that the clone #4-15 has a single exogenous cassette in the desired locus without random integration.

**Figure 7:**
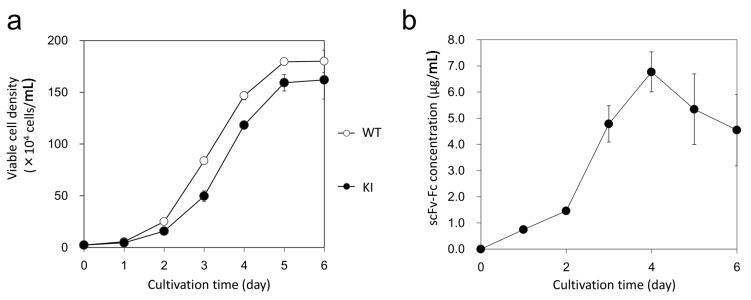
Cell growth and production of scFv-Fc. (**a**) Growth curves of wild-type (WT) and knocked-in (KI) cells. The clone #4-15 was used as the KI cells. Data are expressed as means ± SEM (*n* = 3); (**b**) The production rate of recombinant scFv-Fc in the clone #4-15. Data are expressed as means ± SEM (*n* = 3).

Next, we evaluated the productivity of the clone #4-15 through time-course analyses of cell growth and expression levels of recombinant scFv-Fc. Compared with the wild-type CHO-K1 cells, the clone #4-15 showed slightly slower cell growth (Figure 7a). This difference is quite reasonable, because some of the energy for cell growth have to be spent for the production of recombinant proteins in the clone #4-15. Regarding the ability of scFv-Fc production, the highest concentration of the scFv-Fc protein was observed in day 4 (6.77 ± 0.76 μg/mL), and the maximal productivity in one cell for one day was calculated as 17.8 ± 0.49 pg (Figure 7b). Thus, the clone #4-15 was confirmed to be a good producer cell for recombinant scFv-Fc.

### 2.4. Off-Target Analysis of Knocked-in Cell Clone

We finally analyzed whether off-target mutations were induced in the knocked-in cell clone. We searched the potential off-target sites in the Chinese hamster genome using an online Paired Target Finder tool (see Experimental Section for details). We calculated an average score for each potential off-target site and identified top seven candidate sites (Table 2). These seven loci were amplified by PCR and sequenced using the genomic DNA extracted from backbone-free knock-in cell clone, #4-15. We detected no mutations in all seven loci, suggesting low possibility of off-target mutations in this knock-in clone. Further assessments are needed to completely prove the existence or non-existence of off-target mutations.

## 3. Experimental Section

### 3.1. Construction of TALEN Plasmids and PITCh Vectors

TALEN plasmids were constructed using the Platinum Gate TALEN Kit (Kit # 1000000043, Addgene, Cambridge, MA, USA), according to the previous reports [24,25]. Briefly, DNA-binding domains of TALENs were assembled using the two-step Golden Gate cloning method. ptCMV-153/47-VR vectors were used as destination vectors. For the construction of PITCh vectors, pBApo-EF1α Pur vector (TAKARA BIO INC.) was used as a backbone, and *DsRed* cDNA derived from pIRES2-DsRed-Express2 (Clontech, Palo Alto, CA, USA), TALEN target sites, and an *scFv-Fc* cassette [26] were added to the vector using standard molecular biology methods. The TALEN plasmids used in this study are going to be available from Addgene (ptCMV-CHO-HPRT1-TALEN-L and ptCMV-CHO-HPRT1-TALEN-R).

**Table 2:** Summary of the off-target analysis.

Name	Sequence *	Reference	TAL1 Score **	TAL2 Score **	Average Score	Mutation
On-target	5ʹ-TGAACCAGGCTATGACCTagatttattttgtatTCCTAATCACTATGTCGA-3ʹ 3ʹ-ACTTGGTCCGATACTGGAtctaaataaaacataAGGATTAGTGATACAGCT-5ʹ	gi|351517617|ref|NW_003613932.1|	5.79	6.61	6.2	-
OT1	5ʹ-$\text{T}\underset{-}{\text{A}}\text{AA}\underset{-}{\text{G}}\text{CAGGCTATGACCT}$tttccaactggaattttctccaaccAGGTGAGTGTTTGATTTA-3ʹ 3ʹ-ATTTCGTCCGATACTGGAaaaggttgaccttaaaagaggttgg$\text{TCCA}\underset{-}{\text{C}}\text{T}\underset{-}{\text{CA}}\text{C}\underset{-}{\text{AA}}\text{AC}\underset{-}{\text{T}}\text{AA}\underset{-}{\text{A}}\text{T}$-5ʹ	gi|351517757|ref|NW_003613792.1|	9.53	14.74	12.135	Not detected
OT2	5ʹ-$\text{T}\underset{-}{\text{AA}}\text{ACA}\underset{-}{\text{C}}\text{AGTGA}\underset{-}{\text{CC}}\text{A}\underset{-}{\text{AA}}\text{A}$gcaacttaggagaaaAGGGTATATTTTGGTTTA-3ʹ 3ʹ-ATTTGTGTCACTGGTTTTcgttgaatcctcttt$\text{TCC}\underset{-}{\text{CA}}\text{TAT}\underset{-}{\text{AAA}}\text{ACCAA}\underset{-}{\text{A}}\text{T}$-5ʹ	gi|351517841|ref|NW_003613708.1|	15.55	10.96	13.255	Not detected
OT3	5ʹ-$\text{T}\underset{-}{\text{A}}\text{AACCAGGCTAT}\underset{-}{\text{A}}\text{A}\underset{-}{\text{GA}}\text{T}$ccaacagtgagaatattaaaaTAAAAATTACTATGTTGA-3ʹ 3ʹ-ATTTGGTCCGATATTCTAggttgtcactcttataatttt$\text{A}\underset{-}{\text{TTT}}\text{TTA}\underset{-}{\text{A}}\text{TGATACA}\underset{-}{\text{A}}\text{CT}$-5ʹ	gi|351517438|ref|NW_003614111.1|	12.05	14.61	13.33	Not detected
OT4	5ʹ-$\text{TC}\underset{-}{\text{C}}\text{AC}\underset{-}{\text{CA}}\text{AG}\underset{-}{\text{CC}}\text{ATTAGGA}$cagattccttttgtTCCTGATGACTAGGTTGA-3ʹ 3ʹ-AGGTGGTTCGGTAATCCTgtctaaggaaaaca$\text{AGGA}\underset{-}{\text{C}}\text{TA}\underset{-}{\text{C}}\text{TGAT}\underset{-}{\text{C}}\text{CA}\underset{-}{\text{A}}\text{CT}$-5ʹ	gi|351516302|ref|NW_003615247.1|	14.78	11.91	13.345	Not detected
OT5	5ʹ-$\text{TC}\underset{-}{\text{A}}\text{A}\underset{-}{\text{A}}\text{A}\underset{-}{\text{A}}\text{A}\underset{-}{\text{A}}\text{T}\underset{-}{\text{A}}\text{A}\underset{-}{\text{A}}\text{TA}\underset{-}{\text{AA}}\text{A}$gttaattaattaattaaatcaatacaaatTTATATTCATTATGTAGA-3ʹ 3ʹ-AGTTTTTTTATTTATTTTcaattaattaattaatttagttatgttta$\text{A}\underset{-}{\text{AT}}\text{AT}\underset{-}{\text{A}}\text{AGT}\underset{-}{\text{A}}\text{ATACA}\underset{-}{\text{T}}\text{CT}$-5ʹ	gi|351517764|ref|NW_003613785.1|	13.95	13.1	13.525	Not detected
OT6	5ʹ-$\text{T}\underset{-}{\text{AT}}\text{ACATA}\underset{-}{\text{A}}\text{T}\underset{-}{\text{A}}\text{A}\underset{-}{\text{A}}\text{TA}\underset{-}{\text{AAT}}$aaatctttaaaaaaacaaAAGTTATAGTTTGGTTTA-3ʹ 3ʹ-ATATGTATTATTTATTTAtttagaaatttttttgtt$\text{T}\underset{-}{\text{T}}\text{CA}\underset{-}{\text{A}}\text{TATC}\underset{-}{\text{AA}}\text{ACCAA}\underset{-}{\text{A}}\text{T}$-5ʹ	gi|351516670|ref|NW_003614879.1|	17.3	9.84	13.57	Not detected
OT7	5ʹ-$\text{T}\underset{-}{\text{AT}}\text{ACATA}\underset{-}{\text{A}}\text{T}\underset{-}{\text{A}}\text{A}\underset{-}{\text{A}}\text{TA}\underset{-}{\text{AAT}}$aaataaataaatctttttaaaAAGTTATAGTTTGGTTTA-3ʹ 3ʹ-ATATGTATTATTTATTTAtttatttatttagaaaaattt$\text{T}\underset{-}{\text{T}}\text{CA}\underset{-}{\text{A}}\text{TATC}\underset{-}{\text{AA}}\text{ACCAA}\underset{-}{\text{A}}\text{T}$-5ʹ	gi|351517952|ref|NW_003613597.1|	17.3	9.84	13.57	Not detected

* Red and blue letters indicate the left and right TALEN target sequences, respectively. Lowercase letters indicate spacer sequence. Mismatches are underlined; ** TAL1 and TAL2 scores were calculated by the Paired Target Finder tool [27].

### 3.2. Cell Culture and Transfection

CHO-K1 cells were obtained from RIKEN Cell Bank (Tsukuba, Japan) and maintained in Ham’s F12 medium supplemented with 10% fetal bovine serum. For the genomic cleavage detection assay, 2 × 10^5^ CHO-K1 cells were seeded in 35-mm dishes a day before transfection. The transfection was carried out with or without 1.25 μg each of the left and right TALEN plasmids using Lipofectamine LTX (Life Technologies, Carlsbad, CA, USA), according to the manufacturer’s instruction. For gene knock-in, 1 × 10^5^ CHO-K1 cells were seeded in 100-mm dishes a day before transfection. The transfection was carried out with 0.8 μg each of the PITCh vector and the left and right TALEN plasmids using Lipofectamine LTX (Life Technologies).

### 3.3. Genomic Cleavage Detection Assay

The genomic cleavage detection assay was performed as previously described [28] with some modifications. Briefly, the transfected cells were cultured for three days, and then collected in microtubes. Genomic DNA was extracted using a DNeasy Blood & Tissue Kit (QIAGEN, Hilden, Germany). PCR amplification, denaturation and re-annealing, and genomic cleavage detection assay were carried out using a GeneArt Genomic Cleavage Detection Kit (Life Technologies), according to the manufacturer’s instruction. Primers used in this assay were listed in Table 3.

### 3.4. Puromycin Selection and Single Cell Cloning

Puromycin selection and single cell cloning were performed as previously described [8] with modifications. Briefly, selection was started at 24 or 72 h post-transfection, by supplementing with 50 μg/mL puromycin. The culture media was replaced with a fresh media containing puromycin on a daily basis. After around one week of puromycin selection, single cell cloning was performed using a limiting dilution method with a 96-well plate according to the following protocol. The cells were trypsinized, collected, and adjusted to 7.5 cells/mL. Subsequently, 200 μL of the suspended cells was moved to each well of a 96-well plate (1.5 cells/well).

### 3.5. Fluorescence Microscopy

Fluorescence was observed around 1–1.5 week after single cell cloning. The bright-field and fluorescence images were obtained using fluorescence microscopes (CKX41 or IX81; Olympus, Tokyo, Japan).

### 3.6. Genotyping and Sequencing

Genomic DNA was extracted using a DNeasy Blood & Tissue Kit (QIAGEN). After PCR genotyping using primers listed in Table 3, PCR products of 5′ and 3′ junctions from the correctly knocked-in cell clones were directly sequenced using an ABI 3130xl Genetic analyzer (Life Technologies) with a BigDye Terminator v3.1 Cycle Sequencing Kit (Life Technologies).

### 3.7. Southern Blotting

Southern blotting was performed according to the previously described protocol [8] with modifications. Briefly, 10-μg aliquots of genomic DNA were digested with HindIII and KpnI, and 1-μg aliquots of the digested genome were resolved on a 1% agarose gel. Digoxigenin-labeled DNA probes were made by PCR using Ex *Taq* (TAKARA BIO INC.) and DIG DNA labeling mix (Roche, Basel, Switzerland) with primers listed in Table 3. Membrane transfer (Hybond-N+; GE Healthcare, Waukesha, WI, USA), UV cross-linking (120 mJ/cm^2^), pre-hybridization and hybridization were performed according to the instructions for DIG Easy Hyb Granules (Roche). The CSPD, ready-to-use (Roche) was used to develop the membrane, following the manufacturer’s instructions. The chemiluminescent signal was detected using an X-ray film (RX-U; Fuji, Kanagawa, Japan).

**Table 3:** Primers used in this study.

Genomic Cleavage Detection Assay		
Locus	Forward primer (5′→3′)	Reverse primer (5′→3′)
*HPRT1*	GCCCTTCATACCCTCTCATACCC	GCCATCTCTCCAGCCCCTTC
**Genotyping of Knock-In Clones**		
Locus	Forward primer (5′→3′)	Reverse primer (5′→3′)
5′ junction (in 2.2.1.)	GCATTTGCCCCCACAATGCTC	AGGCGGAGCCAGTACACGACATC
3′ junction (in 2.2.1.)	GGTGCCTGAAGATCCAGACATGATAAG	TGTCCCTGCAGGCCAGAAGAG
Non-knock-in (in 2.2.1.)	GCATTTGCCCCCACAATGCTC	TGTCCCTGCAGGCCAGAAGAG
5′ junction (in 2.2.2.)	TCATACCCAAATTCCTCTGGCGAAC	TGTGCGCTCTGCCCACTGAC
3′ junction (in 2.2.2.)	GGTGCCTGAAGATCCAGACATGATAAG	AGCAGTCAGTGCTCTTAACCGCTGAG
Non-knock-in (in 2.2.2.)	TCATACCCAAATTCCTCTGGCGAAC	AGCAGTCAGTGCTCTTAACCGCTGAG
5′ junction (in 2.2.3.)	GCATTTGCCCCCACAATGCTC	GGACTTTCCACACCTGGTTGCTGAC
3′ junction (in 2.2.3.)	AAGCTTGTCGACATCGATGAATCAGG	ACTTCAATTAAGATCTGTTCTGTCTGCATGTGTC
Non-knock-in (in 2.2.3.)	GCATTTGCCCCCACAATGCTC	ACTTCAATTAAGATCTGTTCTGTCTGCATGTGTC
**Southern Blot Analysis**		
Locus	Forward primer (5′→3′)	Reverse primer (5′→3′)
Probe	CCGAGCTGCAAGAACTCTTCCTCAC	GGTCCTTCGGGCACCTCGAC
**Off-Target Analysis**		
Locus	Forward primer (5′→3′)	Reverse primer (5′→3′)
OT1	TCACTAGCAGGTGTGACTCTCAGTACGC	GGAAACCTGAATAGACAGACACAAGCAAG
OT2	CTAATTTGACCTTCCTGTTCACCAGTGCTAC	GATAGCATGAGAAAGCAAACTGAGCAAGC
OT3	CAGTTCCCATGCTTACAGCATCAGTG	CTCATGCCTATGTGGCAAGTGCTTTAC
OT4	GTCCCAAGCCTTTCTGAATTATTTCTACTTCC	AAAGCATCAAAATGTCCTCGCATTGAG
OT5	CACACACCCTCTCACTTTCATTCTCTCTC	ATGAGGGAAGTTTGAAGAGAATAATATGGAAAGG
OT6	GCAATACGGGTGATTGAAGAGCACTG	CTCCTCCAACTTCATTGTACTCTACGCATTC
OT7	CTCTGTAGTTTGGTCTCTGATGGCAGTTTG	CCAAGGAAGCAGTCAGCTCTACCATAAAC

### 3.8. Cell Growth Analysis and Expression Analysis of scFv-Fc

Cell growth and scFv-Fc expression were analyzed as described before [26]. Briefly, 2.5 × 10^4^ cells were seeded in 2 mL of the serum-containing media in 6-well plates and cultured for 6 days. Total volume of the media was replaced with fresh media every day. Viable cell density was determined by the trypan blue exclusion method. A solid-phase enzyme-linked immunosorbent assay (sandwich ELISA) was performed to measure the scFv-Fc concentration as described previously [29,30].

### 3.9. Off-Target Analysis

The potential off-target sites were determined using the Paired Target Finder (https://tale-nt.cac.cornell.edu/node/add/talef-off-paired) [27] with the following settings. The NCBI ID GCA_000223135.1 was used as the reference genome database. The RVD sequences were provided as follows; RVD sequence 1, NN NI NI HD HD NI NN NN HD NG NI NG NN NI HD HD NG; RVD sequence 2, HD NN NI HD NI NG NI NN NG NN NI NG NG NI NN NN NI. Minimum and maximum spacer lengths were set to 10 and 30, respectively. Other parameters were set as recommended. Genomic regions around each candidate site were amplified by PCR using primers listed in Table 3, and the sequence was confirmed by direct sequencing as described above.

## 4. Conclusions

In this study, we first demonstrated the purposeful gene knock-in carrying a productive gene cassette using genome editing technology in CHO cells. Although the knock-in efficiency described herein is necessary and sufficient for isolating a correctly targeted clone(s) with a single transfection and limiting dilution even in nearly 10-kb plasmid integration, there are still many random integrants and partially knocked-in clones. Since we have also succeeded in backbone-free integration with a comparable efficiency, an addition of a negative selection marker such as the *Herpes simplex* virus thymidine kinase to the plasmid backbone might further increase the knock-in efficiency. In addition, MMEJ-directed PITCh strategy did not always result in the expected knock-in junctions, although the subtle mutations affect neither the outcome of biopharmaceuticals’ production nor selection of knock-in cell clones both by antibiotics and fluorescence. Nevertheless, further improvements are required for generating more precise knock-in junctions, because imprecise junction might be a limitation of our method, especially for application with endogenous genetic elements (e.g., fusion to the start codon or to the last codon of the target genes). Another limitation of the TALEN-mediated PITCh strategy is the fixed length of microhomologies due to the limited range of spacer length in the TALEN target site. This problem is thought to be solved by using the CRISPR/Cas9 approach.

As shown in our previous study, CRISPR/Cas9-mediated PITCh can also be applied in mammalian cells and animals [8,9]. In zebrafish, side-by-side comparison of the frequency and accuracy of targeted integration using donor vectors harboring various lengths of microhomologies (0, 10, 20, and 40 bp) revealed that the addition of 10-, 20-, or 40-bp microhomologies increased the frequency of targeted integration, and that the longer microhomologies (20 and 40 bp) resulted in higher accuracy of integration than the shorter one (10 bp) [9]. Although we have chosen TALEN in this study in anticipation of its reportedly high specificity, and the selected potential off-target sites were not mutated, most of the DsRed-positive clones were not the on-target integrants. The CRISPR/Cas9 approach will be an attractive alternative to the TALEN strategy described herein.

Collectively, this study provides highly practical insights and challenges to overcome for animal cell engineering, especially for the production of biopharmaceuticals in CHO cells.
